# The neural encoding of self-generated and externally applied movement: implications for the perception of self-motion and spatial memory

**DOI:** 10.3389/fnint.2013.00108

**Published:** 2014-01-13

**Authors:** Kathleen E. Cullen

**Affiliations:** Aerospace Medical Research Unit, Department of Physiology, McGill UniversityMontreal, QC, Canada

**Keywords:** proprioception, self-motion, head direction cells, place cells, sensory coding, efference copy, corollary discharge, voluntary movement

## Abstract

The vestibular system is vital for maintaining an accurate representation of self-motion. As one moves (or is moved) toward a new place in the environment, signals from the vestibular sensors are relayed to higher-order centers. It is generally assumed the vestibular system provides a veridical representation of head motion to these centers for the perception of self-motion and spatial memory. In support of this idea, evidence from lesion studies suggests that vestibular inputs are required for the directional tuning of head direction cells in the limbic system as well as neurons in areas of multimodal association cortex. However, recent investigations in monkeys and mice challenge the notion that early vestibular pathways encode an absolute representation of head motion. Instead, processing at the first central stage is inherently multimodal. This minireview highlights recent progress that has been made towards understanding how the brain processes and interprets self-motion signals encoded by the vestibular otoliths and semicircular canals during everyday life. The following interrelated questions are considered. What information is available to the higher-order centers that contribute to self-motion perception? How do we distinguish between our own self-generated movements and those of the external world? And lastly, what are the implications of differences in the processing of these active vs. passive movements for spatial memory?

## Functionally analogous cells types in the vestibular pathways of monkey and mouse

The vestibular system provides the brain with information about the motion of the head relative to space and is comprised of two types of sensors: the three semicircular canals, which sense angular rotation in three dimensions and the two otolith organs (the saccule and utricle), which sense linear motion (i.e., gravity and three dimensional translational movement). In turn, the receptor cells of the semicircular canals and otoliths send signals through the vestibular nerve fibers to the vestibular nuclei (VN).

To date, the coding of vestibular information at the level of single vestibular nerve afferents and their target neurons in the VN has been well characterized in alert behaving monkeys. Notably, neurons predominantly encode rotational head velocity and linear head acceleration. Vestibular afferents can be further characterized on the basis of their baseline discharge regularity as regular or irregular (reviewed in Goldberg, 2000; Cullen, 2011). In addition, their target neurons in the VN can be divided into three primary groups on the basis of their sensitivities to applied head motion and eye movements (Cullen and McCrea, 1993; Cullen et al., 1993, and reviewed in Cullen, 2012). Two classes of neurons—each with a specific combination of eye movement and vestibular related responses—are thought to provide the substrate for the generation and adaptation of the vestibulo-ocular reflex. In particular, eye movement related inputs from oculomotor areas of the brainstem (e.g., the nucleus prepositus and reticular formation), the accessory optic system, and the vestibular cerebellum (flocculus and ventral paraflocculus) provide saccade, pursuit and optokinetic–related inputs to both neuron classes. These extravestibular inputs contribute to the control and modulation of both visually-driven eye movements and the vestibulo-ocular reflex. In contrast, a third subgroup of neurons that responds to vestibular stimulation but not eye movements projects both to: (i) the spinal cord and (ii) upstream centers including the thalamus and vestibular cerebellum (reviewed in Cullen, 2012), to ensure the maintenance of posture and accurate perception of self-motion.

**Figure 1:**
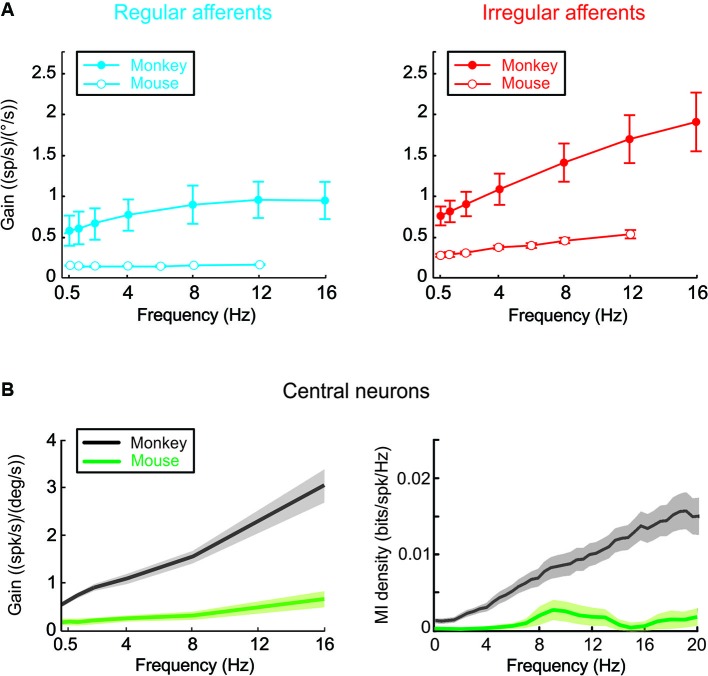
**(A)** Mouse vestibular afferents can be classified on the basis of their discharge regularity, and are on average 3–4 times less sensitive to head velocity when compared to monkey afferents. **(B)** Comparison of rotational sensitivities and mutual information density of mouse and monkey VN neurons. (Monkey data are adapted from Massot et al., 2011, 2012).

More recently, a corresponding series of studies in mice examined the coding of vestibular information at the level of single vestibular nerve afferents and VN neurons. Comparison with findings in monkey reveals that mouse vestibular afferents can likewise be classified on the basis of their discharge regularity, but they are on average 3–4 times less sensitive to head velocity (Figure 1A; Yang and Hullar, 2007; Lasker et al., 2008). Similarly, mouse VN neurons (Beraneck and Cullen, 2007) display relatively low sensitivities to vestibular stimulation as compared to neurons in monkeys (Massot et al., 2011, 2012). Furthermore, simultaneous recordings of eye and head motion responses revealed subgroups comprising both eye motion sensitive and insensitive neurons in the mouse VN similar to those reported in monkey (Beraneck and Cullen, 2007).

Why is early vestibular processing in mice characterized by lower pathway modulation than in monkeys? The general decrease in modulation could potentially indicate sensory processing has adapted to account for differences in the stimuli experienced by each species in its natural environment. Alternatively, it is also possible that neuronal sensitivities are matched to the specific constraints of the reflexes that the mouse sensory-motor pathways evolved to control. If mice have a more limited need for perceptual and behavioral accuracy, then the relatively lower discharges of their early vestibular pathways could correspond to reduced information transmission (Vinje and Gallant, 2000; Borst and Haag, 2001). This proposal is consistent with our preliminary results that in mice eye-movement insensitive neurons encode substantially less information than do monkey VN neurons (Figure 1B; Jamali et al., 2010; Massot et al., 2011). Taken together, current evidence suggests that evolutionary pressure adjusts characteristics of sensory transmission in early vestibular processing to meet certain functional requirements, which differ across species (see also, Niven et al., 2007).

## What information is available to higher-order centers that contribute to the perception of self-motion?

As noted above, vestibular afferents exclusively encode head motion information and project to a class of neurons in the VN that in turn project to both the spinal cord and upstream centers to ensure the maintenance of posture and perception of self-motion. In mice the majority of these VN neurons are sensitive to the dynamic stimulation of neck proprioceptors (Medrea and Cullen, 2013). This finding is consistent with reports that both vestibular and proprioceptive sensory inputs can modulate the response of VN neurons in alert squirrel monkeys (Gdowski and McCrea, 2000) and cynomolgus monkeys (i.e., *Macaca fascicularis*; Sadeghi et al., 2009). In contrast, in alert rhesus monkeys (*Macaca mulatta*), we found that VN neurons do not normally respond to passive stimulation of neck proprioceptors (Roy and Cullen, 2001, 2002, 2003). Instead, such integration is observed only at the next stage in the cerebellum (Brooks and Cullen, 2009, 2013). Interestingly, however, after peripheral vestibular loss, VN neurons in the rhesus monkeys respond to passive proprioceptive stimulation indicating that sensory substitution occurs at the earliest stages of vestibular processing to mediate compensation (Sadeghi et al., 2010, 2011, 2012).

In mice, proprioceptive-related responses can be either “additive” or “subtractive” to vestibular sensitivities. Put another way, they can function to either enhance or reduce vestibular-related modulation when the head is moved relative the animal’s body. This is a condition in which both self-motion sensory cues are present—the vestibular sensors are stimulated by the movement of the head relative to space, while neck proprioceptors are simultaneously activated by the resultant stretch applied the neck. Notably, in mice a given neuron’s response to such combined stimulation can be well predicted by the simple linear sum of its response to each stimulus when applied alone, consistent with previous studies in alert squirrel and cynomolgus monkeys (Gdowski et al., 2001; Sadeghi et al., 2009).

Neck sensitive VN neurons also encode a static neck position signal in alert mice (Medrea and Cullen, 2013) as well as in rats (e.g., Barresi et al., 2013) but not in primates. As detailed below, this static signal of proprioceptive origin is observed during both active and passive self-motion. Thus, this input is of particular interest since it can potentially provide an important heading signal to upstream structures for the computation of spatial orientation.

The lack of a static head position signal in primates as compared to rodents may reflect differences in the active control of gaze as well as habitat. Monkeys, are frontal-eyed animals with a retina specialized for high-acuity vision (fovea). In particular, monkeys often use voluntary coordinated eye-head and eye-head-body gaze shifts (McCluskey and Cullen, 2007) to precisely align gaze when exploring their environment, whereas mice are afoveates for which head and body motion are typically more closely linked during exploration (see Stahl et al., 2006). It is thus likely that the static neck sensitivity coded by mouse VN neurons plays a vital role in stabilization of the head relative to the body during exploration via the vestibulo-collic reflex (e.g., Baker, 2005; Takemura and King, 2005). In contrast, such default stabilization would be potentially detrimental in monkeys, since it would be counterproductive to the voluntary head movements that are frequently made by this species.

## How do we distinguish between our own self-generated movements and those of the external world?

Voluntary neck movements generate egocentric motor-related as well as proprioceptive signals. In the murine vestibular nucleus, these signals are combined with allocentric vestibular signals (head motion in space) at the neuronal level. Specifically, the simple linear summation of a neuron’s sensitivities to passive vestibular and neck proprioceptive stimulation applied alone no longer predicts VN neurons responses (Medrea and Cullen, 2013). Instead, neuronal responses are suppressed for self-generated head motion in a manner similar to what has been observed in monkey (Gdowski and McCrea, 2000; Roy and Cullen, 2001; Sadeghi et al., 2009). Evidence from experiments in monkeys suggest that a neural copy of the motor command that initiates the active motion, is used to cancel self-generated sensory input during active head movements (e.g., Roy and Cullen, 2004; Sadeghi et al., 2009). A comparable mechanism may underlie the analogous suppression of self-produced vestibular stimulation observed in the VN of mice.

It is notable, that a series of lesion and inactivation studies has provided evidence that vestibular inputs are essential to ensure the tuning of the head-direction cell network. Head direction cells are thought to integrate signals of vestibular origin to maintain a signal of cumulative rotation (reviewed in Taube, 2007) and are found in many brain areas, including the post-subiculum, retrosplenial cortex, thalamus, lateral mammillary nucleus, dorsal tegmental nucleus, striatum and entorhinal cortex. A characteristic of head direction cells is that they selectively fire when animal’s head points in a specific direction. Based on anatomical studies, it has been further suggested that three nuclei could relay vestibular signals to the head-direction pathway including: the nucleus prepositus, the supragenual nucleus, and the paragigantocellular nucleus (reviewed in Shinder and Taube, 2011). However, at least in monkeys, the nucleus prepositus predominantly encodes eye-movement information during both externally applied and passively generated motion (Dale and Cullen, 2013). On the other hand, lesions to the supragenual nucleus can destabilize head-direction cell tuning in rats (Clark and Taube, 2012). Future neurophysiological experiments in mice and rats quantifying both vestibular and extravestibular related responses during self-motion will be needed to fully understand the nature of the signals that these nuclei relay to upstream structures.

## What are the implications regarding the neural encoding of self-generated vs. externally applied motion for spatial memory?

**Figure 2:**
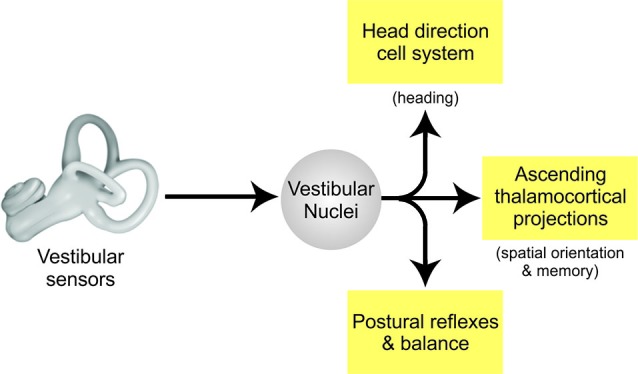
**Vestibular pathways for spatial memory.** Vestibular signals from the labyrinth of the inner ear are transferred to the VN via the vestibular afferents of the VIII nerve. In turn, the VN projects to other brain areas to: (i) control posture and balance, (ii) produce estimates of spatial orientation, and (iii) encode heading direction.

What is the functional significance of the differential encoding of active and passive motion by early vestibular pathways in mice? Three important implications are outlined below (Figure 2).

First, the descending projections of this specific class of VN neurons mediate spinal postural reflexes such as the vestibulo-collic reflex (Wilson et al., 1990; Boyle et al., 1996). Thus, the fact that sensory inputs produced by volitional movement are suppressed suggests that these stabilizing reflex pathways are themselves suppressed. This is helpful, since an intact reflex command would be counterproductive to the intended movements when the behavioral goal is to generate active self-motion.

Second, the multimodal information encoded by the ascending thalamocortical projections of this specific class of VN neurons make a major contribution to higher-level functions including the computation for spatial orientation and memory. Two facts discussed above play together. On the one hand multimodal integration in the VN is more comprehensive in mice than in monkeys. On the other hand, we found that monkeys might implement some functionality found in the murine VN in the cerebellar cortex instead (Brooks and Cullen, 2009, 2013).

Third and finally, recent neurophysiological findings have specific implications for the head direction cell network. While this network is commonly thought to integrate signals of vestibular origin to maintain a signal of cumulative rotation (reviewed in Taube, 2007), the neurophysiological studies reviewed above have established that the coding of vestibular information at the first central stage of processing is determined by on-going behavior in both primates and rodents. In particular, vestibular information is combined with egocentric information including proprioceptive and motor-related signals at this initial stage of sensory processing. One possibility is that egocentric cues provided by the proprioceptive and motor-related signals in early vestibular processing also make important contributions to the head direction cell network activity (Wiener et al., 2002; Taube and Basset, 2003). Notably, the observed motor-related responses could potentially provide a directional heading signal with anticipatory features. Furthermore, it is likely that different species employ characteristic integration strategies, based on specific weighting of egocentric as well as allocentric cues, to compute the head direction signal. For instance, Yoder and Taube (2009) reported that rat head direction cells are more influenced by external (i.e., visual) cues than those of mice. Future work is needed to fully understand the contribution of the egocentric signals encoded by vestibular pathways to head direction cell signal generation and whether there are important differences in this computation across species (e.g., mouse vs. rat vs. monkey). This knowledge will be essential in furthering our understanding of how input pathways such as the early vestibular system that encode both allocentric and egocentric information, contribute to the neural representation of direction encoded by higher level structures.

## Conclusion

It is generally assumed the vestibular system provides a veridical representation of head motion to higher order centers for the perception of self-motion and spatial orientation. However, as reviewed above, the findings of recent electrophysiological studies in monkeys and mice have challenged this assumption. Instead, under natural conditions, behavioral context governs how vestibular information is encoded at the first central stage of vestibular processing. Not only is processing inherently multimodal, but the manner in which multiple inputs are combined is adjusted to meet the needs of the current behavioral goal. Notably, in natural conditions, neurons in the VN can distinguish between active and passive motion—responding far more robustly to passive movements. These results have important implications for understanding the computations that underlie the spatial orientation signals encoded by neurons in the head direction cell network and areas of multimodal association cortex that underlie self-motion perception and spatial memory.

## Conflict of interest statement

The author declares that the research was conducted in the absence of any commercial or financial relationships that could be construed as a potential conflict of interest.
